# Bioinformatics analysis for the identification of Sprouty-related EVH1 domain-containing protein 3 expression and its clinical significance in thyroid carcinoma

**DOI:** 10.1038/s41598-024-55187-2

**Published:** 2024-02-24

**Authors:** Xiaowei Zhang, Xiangwei Meng, Pengyun Wang, Chong Luan, Haiming Wang

**Affiliations:** 1 Department of Orthopedics, Zibo Central Hospital, No 54, Gong Qing Tuan Xi Road, Zibo, 255036 People’s Republic of China; 2 Department of Drug Clinical Trials, Zibo Central Hospital, Zibo, People’s Republic of China; 3 Department of thyroid and breast surgery, Zibo Municipal Hospital, Zibo, 255400 People’s Republic of China

**Keywords:** Cancer, Computational biology and bioinformatics

## Abstract

The poorly differentiated thyroid carcinoma (THCA) subtype is associated with an aggressive disease course, a less favorable overall prognosis, and an increased risk of distant organ metastasis. In this study, our objective was to explore the potential utility of the Sprouty-related EVH1 domain-containing protein 3 (SPRED3) as a biomarker for early diagnosis and prognosis in THCA patients. The differentially expressed prognostic-related genes associated with THCA were identified by querying The Cancer Genome Atlas (TCGA) database. The difference in the expression of the SPRED3 gene between thyroid carcinoma (THCA) tissues and normal tissues was analyzed using data from The Cancer Genome Atlas (TCGA) and further validated through immunohistochemistry. Univariate and multivariate Cox regression models were used, along with clinical information from THCA patients, to analyze the prognostic value of the SPRED3 gene in THCA patients. Functional enrichment analysis was subsequently performed to elucidate the molecular mechanisms underlying the regulatory effects of the SPRED3 gene on thyroid carcinoma. Additionally, we calculated the percentage of infiltrating immune cells in THCA patients and evaluated their correlation with SPRED3 gene expression. Compared with those in noncancerous thyroid tissue, the gene and protein expression levels of SPRED3 were found to be elevated in thyroid carcinoma tissues. Furthermore, the expression of SPRED3 in thyroid carcinoma exhibited significant correlations with tumor location, histological grade, pathological stage, and tumor node metastasis classification (TNM) stage. Univariate and multivariate Cox proportional hazards (Cox) regression analyses demonstrated that SPRED3 could serve as an independent prognostic factor for predicting the overall survival of THCA patients. The results of functional enrichment analysis suggested the potential involvement of SPRED3 in the regulation of extracellular matrix organization, epidermal development, signaling receptor activator activity, skin development, receptor ligand activity, glycosaminoglycan binding, neuroactive ligand‒receptor interaction, the IL-17 signaling pathway, and the PI3K-Akt signaling pathway. Additionally, there were significant correlations between the expression level of the SPRED3 gene and the infiltration of various immune cells (eosinophils, central memory T cells, neutrophils, macrophages, and NK cells) within the thyroid tumor microenvironment. SPRED3 can be used as a prognostic biomarker in patients with THCA could potentially be therapeutic target for THCA.

## Introduction

Thyroid carcinoma (THCA) is a malignant neoplasm originating from the follicular or parafollicular epithelial cells of the thyroid gland and represents the most prevalent malignancy in the head and neck region^1^. In recent years, the global incidence of differentiated thyroid carcinoma (DTC) has rapidly increased^2^. THCA, particularly the differentiated subtype, accounts for more than 90% of all cases and has a favorable prognosis, with a 10-year survival rate exceeding 95%^3^. However, despite the generally positive outlook for most DTC patients, there is a subset of patients that have a more heterogenous and aggressive variant of THCA that is prone to metastasis and recurrence, thereby impacting subsequent treatment strategies and overall survival rates^4,5^. Moreover, numerous patients are precluded from undergoing surgical intervention for various reasons, significantly compromising their chances of survival^6,7^. The inadequate or unsatisfactory management of this patient subset can be attributed to limited research on the potential mechanisms underlying the invasiveness of this THCA variant, particularly in terms of immune regulation involving specific genes^8^. In recent years, substantial advancements have been made in understanding the potential molecular pathways affected by THCA^9^. Along with epigenetic changes such as abnormal gene methylation, gene mutations, including BRAF, RAS, PIK3CA, PTEN, TP53 and β-catenin mutations, play crucial roles in initiating THCA^10,11^. The molecular pathogenesis of THCA involves aberrant activation of various signaling pathways, such as the mitogen-activated protein kinase (MAPK), phosphatidylinositol 3-kinase/protein kinase B (PI3K/AKT), and nuclear factor-kB (NF-kB) signaling pathways^12^. With extensive investigations into the molecular pathogenesis of THCA, biological targeted therapy is gradually being implemented in clinical practice^13^. There is an urgent need for new biomarkers to aid the early detection and effective clinical treatment of THCA patients.

In this study, we identified Sprouty-related, EVH1 domain-containing protein 3 (SPRED3), which is highly expressed in THCA. Additionally, we explored the potential of SPRED3 as an early diagnostic and prognostic marker of THCA. Notably, SPRED (Sprouty-related proteins with an EVH1 domain) is a recently discovered family of membrane proteins^14^. The mammalian SPRED family comprises four members, namely, SPRED1, SPRED2, SPRED3, and EVE-3, the latter being a spliced variant of SPRED3^15^. Typically, the SPRED protein contains three domains: the N-terminal Enabled/VASP homology 1 (EVH1) domain, the central c-Kit binding domain (KBD), and the C-terminal Sprouty-related domain (SPR)^16^. Research has demonstrated that the SPRED protein family negatively regulates the ERK/MAPK signal transduction pathway through its KBD domain. This modulation influences cellular growth; development; proliferation; angiogenesis; angiogenesis; lymphangiogenesis; osteogenesis; and tissue motility. Moreover, it is closely associated with tumor cell occurrence, progression, migration and invasion^17,18^. The expression levels of SPRED1 and SPRED2 in tumor tissues are lower than those in normal tissues, suggesting that the downregulation of SPRED1 and SPRED2 may serve as important indicators of tumor initiation and progression^19^. Investigating the role of SPREDs could reveal novel therapeutic strategies and targets for cancer treatment. IT has been recently revealed that mutations resulting in the loss of SPRED1 function occur in ∼ 2% of human cancers, and genetic removal of SPREDs leads to various phenotypes, such as behavioral issues, dwarfism, and increased susceptibility to leukemia in mice^18^. Recent research has revealed that a loss-of-function mutation in SPRED3 is responsible for activating the Ras/Raf/MAPK pathway in non-small cell lung cancer (NSCLC) cells^20^. However, no reports have been published regarding the expression of SPRED3 in THCA thus far.

The objective of this study was to establish the diagnostic value of SPRED3 expression in patients with thyroid cancer (THCA) and its molecular function, as well as its association with immune infiltration. The TCGA dataset was utilized to gather information on THCA patients and assess the molecular function of SPRED3 as an early diagnostic and prognostic marker. Immunohistochemistry was performed to detect the expression of SPRED3 in THCA cells and adjacent noncancerous tissues, the results of which validated the findings obtained from gene sequencing.

## Results

### Identification of differentially expressed prognostic-related genes in THCA patients

**Figure 1:**
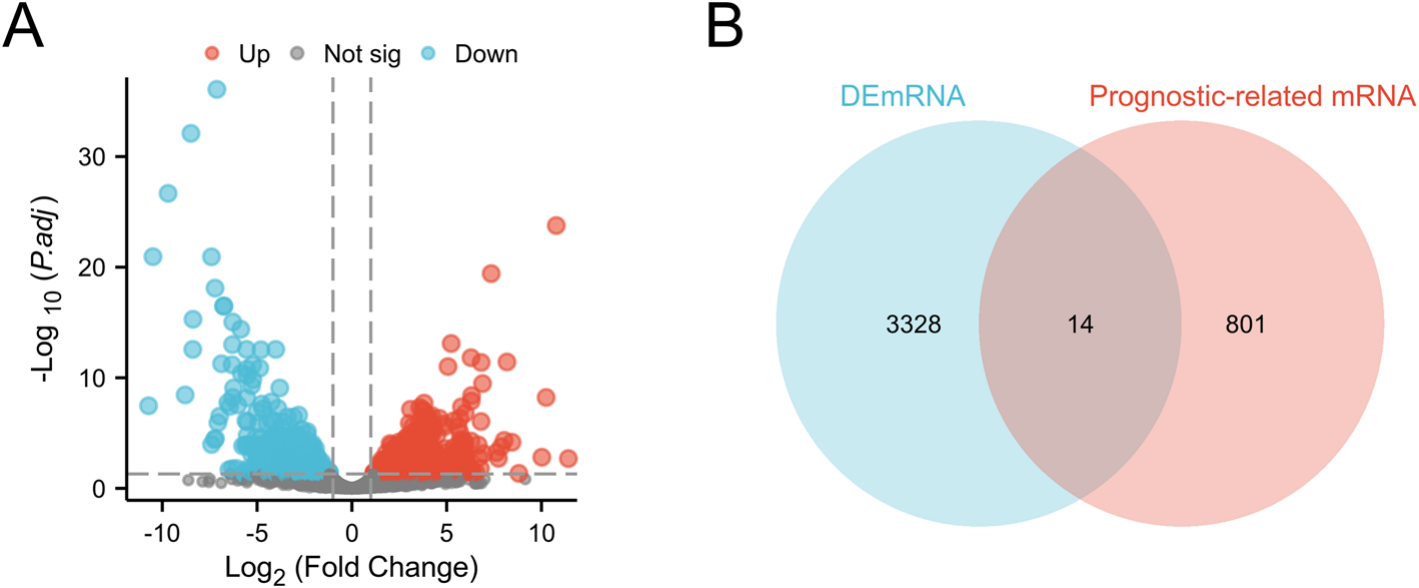
The identification of differentially expressed mRNA associated with prognosis in thyroid carcinoma, showcasing (**A**) a volcanic map depicting the differential expression pattern of mRNA, and (**B**) a Venn diagram highlighting the overlap between differentially expressed mRNA and genes related to prognosis.

In our study, we identified DEGs in 571 thyroid cancer tissues compared to 59 paracancerous tissues by querying the TCGA database. (Fig. 1A). Furthermore, the genes associated with the prognosis of THCA patients were determined through an analysis of prognostic data from the TCGA, and the prognosis-related genes and differentially expressed genes were compared to identify the shared genes. Our data indicated that the upregulation of 14 genes (including OTX1, SYT5, DMRT1, FCRLB, SMTNL2, SPRED3, FAM155A, PCDHA5, PPP1R3G, RPL13AP12, CRNDE, THBS4-AS1, AC008875.1 and AL445649.1) was associated with poor prognosis in patients with THCA (Fig. 1B).

### The expression level of SPRED3 has exceptional diagnostic value for patients with THCA

**Figure 2:**
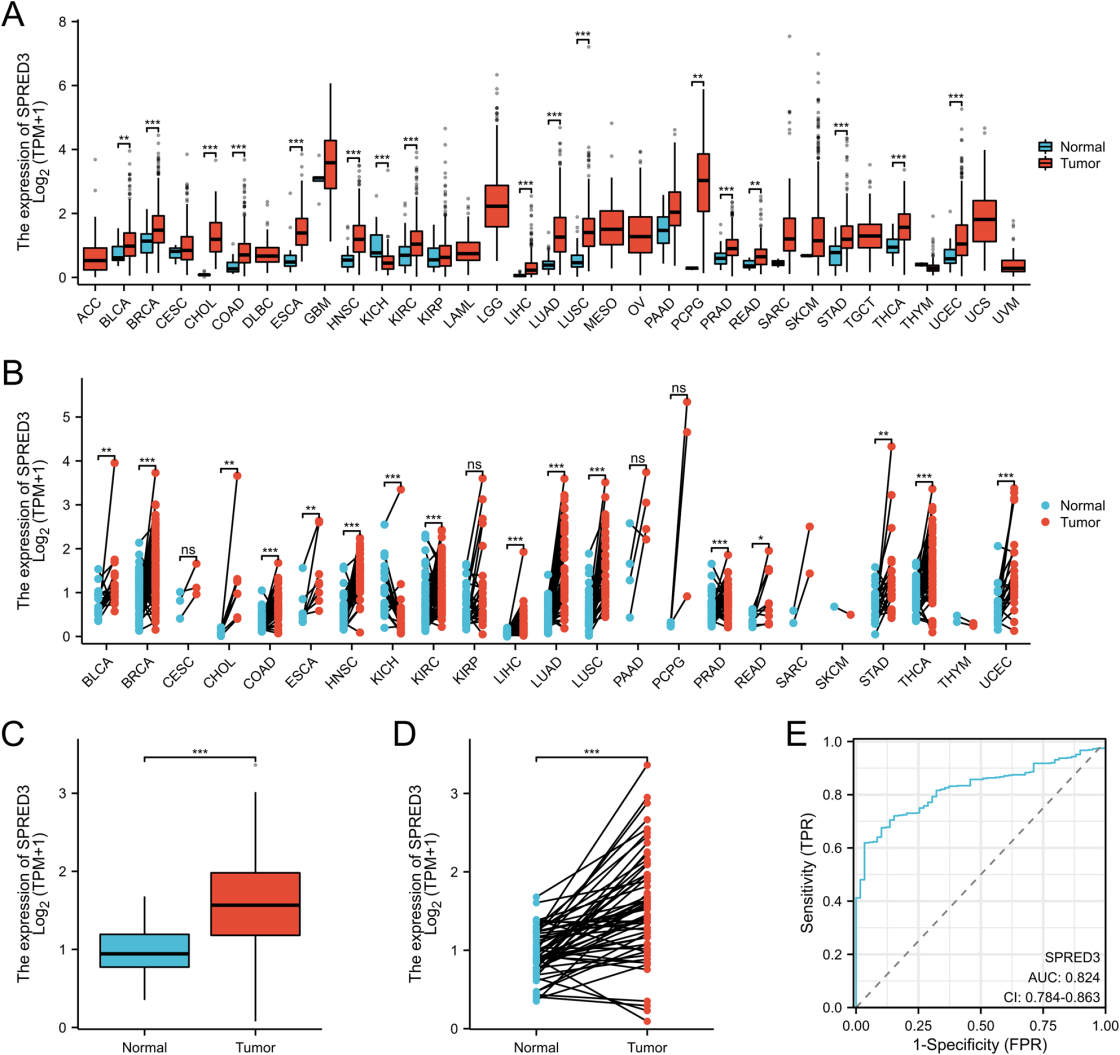
The expression level of SPRED3 is evaluated and exhibits exceptional diagnostic predictive value for patients with THCA. (**A**) Comparative analysis of SPRED3 mRNA expression levels in tumor and adjacent normal tissues across multiple malignancies. (**B**) The SPRED3 mRNA levels were quantified using RNA-seq data obtained from tumor samples and their matched normal tissues in the TCGA database. (**C**) Analysis of sequencing data revealing differential expression of SPRED3 mRNA between normal and THCA (thyroid carcinoma) tissues. (**D**) Validation of SPRED3 mRNA expression through RNA sequencing in paired samples of THCA and normal thyroid tissues, utilizing publicly available GTEx platform data. (**E**) ROC curve illustrating the diagnostic value assessment of SPRED3 expression in THCA samples.

Given the potential to uncover novel therapeutic strategies and targets for cancer treatment through investigating the role of SPREDs in THCA, RNAseq data from 33 tumor studies were retrieved from The Cancer Genome Atlas (TCGA) database and organized, with TPM-formatted data extracted following the STAR process. The mRNA levels of SPRED3 in various malignant cancer types were determined using the Wilcoxon rank sum test. The analysis revealed that SPRED3 exhibited significantly greater expression in various cancer types, including THCA (thyroid carcinoma), UCEC (endometrial cancer), STAD (gastric adenocarcinoma), LUAD (lung adenocarcinoma), BLCA (bladder urothelial carcinoma), BRCA (breast infiltrating carcinoma), CESC (cervical squamous and adenocarcinoma), CHOL (bile duct carcinoma), COAD (colon carcinoma), ESCA (esophageal carcinoma), GBM (glioblastoma), HNSC (head and neck squamous cell carcinoma), KIRC (kidney clear cell carcinoma), LUSC (lung squamous cell carcinoma), PAAD (pancreatic cancer) and PRAD (prostate cancer), than in both normal tissues and the corresponding adjacent normal tissue samples (Fig. 2A,B). Our data revealed that the expression pattern of PPP1R3G varies across different types of cancer. Conversely, SPRED3 was found to be expressed at low levels in renal chromophobe cell carcinoma (KICH) cells (Fig. 2A,B). In addition, through querying the TCGA database, we revealed that the expression level of SPRED3 in THCA tissues (n = 571) was significantly greater than that in normal thyroid tissues (n = 59) (Fig. 2C). Additionally, we retrieved paired para-cancerous normal tissue samples from the TCGA database for further analysis. We conducted a comparative examination of SPRED3 expression in tumor tissue and corresponding paracancerous normal thyroid tissue from THCA patients (n = 58). The results showed that SPRED3 expression was significantly upregulated in thyroid carcinoma tissues (Fig. 2D). Subsequently, the predictive value of the SPRED3 expression level for diagnosis was assessed using receiver operating characteristic (ROC) curve analysis. The receiver operating characteristic (ROC) analysis findings indicated that SPRED3 expression is a strong discriminative factor. The results demonstrated an area under the curve (AUC) of 0.824 for SPRED3 expression in THCA tissues compared to that in normal tissues (Fig. 2E), further supporting its excellent diagnostic predictive value in patients with THCA.

### SPRED3 expression was significantly correlated with tumor location, histological grade and TNM stage in THCA patients

**Figure 3:**
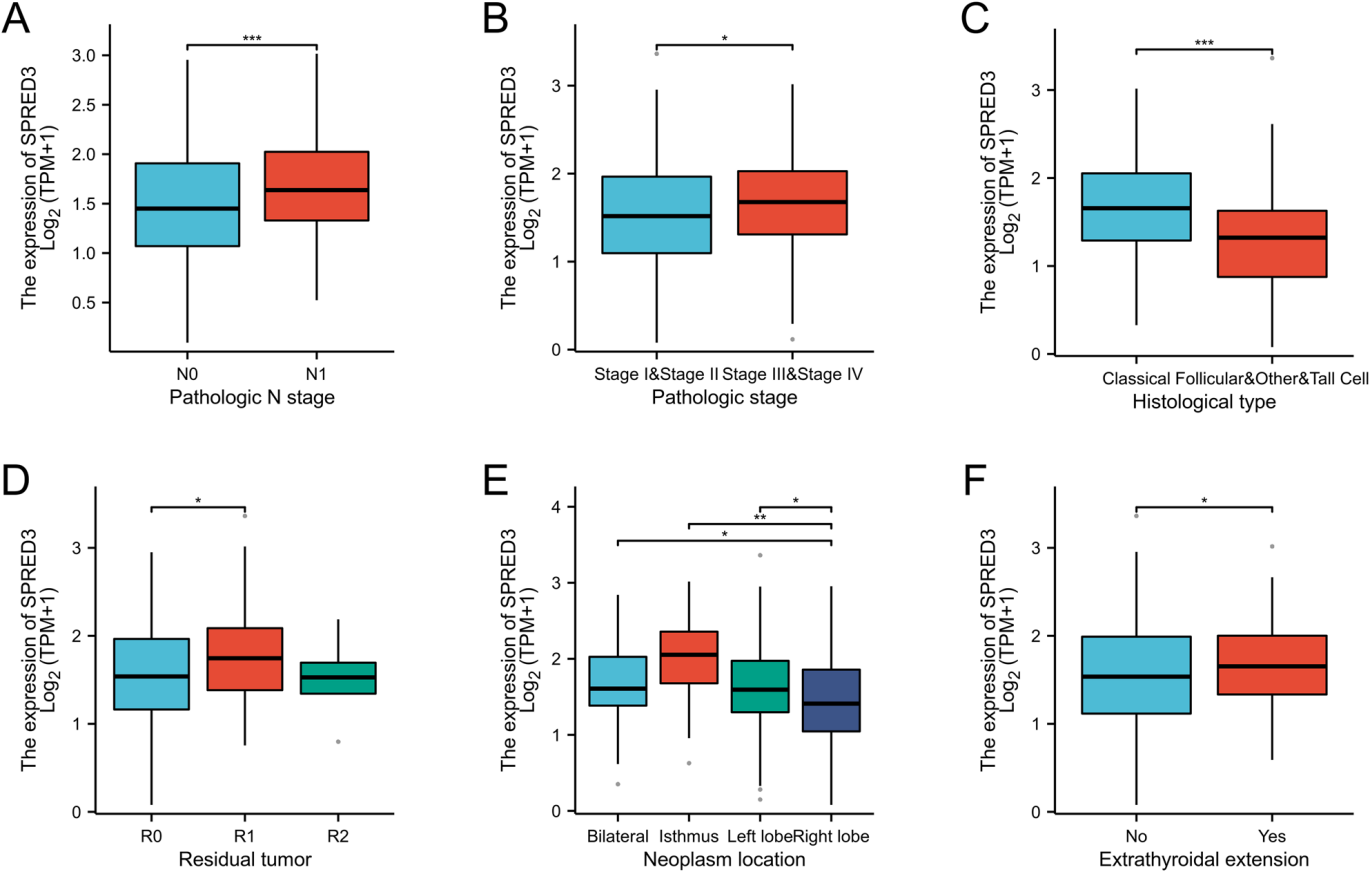
Box plot evaluating SPRED3 mRNA expression differences among THCA patients with different clinical characteristics including disease stages (pathologic N stage) (**A**), tumor pathologic stages (**B**), histologic types (**C**), residual tumor (**D**), neoplasm location (**E**), and the state of extra-thyroidal extension (**F**).

By analyzing the TCGA database and effectively organizing the RNAseq and clinical data from THCA studies in the TCGA, our findings revealed significant upregulation of SPRED3 expression in the N1-stage group compared to the N0-stage group (Fig. 3A). Moreover, there was a significantly greater expression level in the advanced pathological stage III-IV subgroup than in the stage I-II subgroup (Fig. 3B), and the classical subtype exhibited a significantly greater expression level than the nonclassical (follicular type, high-cell type) subtype (Fig. 3C). Additionally, we observed a significant increase in the expression of these genes in patients with residual tumor (R1) compared to those without any remaining lesion (R0) (Fig. 3D). Furthermore, isthmus tumors displayed notably higher levels of SPRED3 expression than other subtypes (Fig. 3E). Finally, there was a significantly greater expression level in patients with extrathyroid extension than in those without extension beyond the thyroid gland boundaries (Fig. 3F).

### High SPRED3 expression predicts a significantly lower overall survival rate in THCA patients

**Figure 4:**
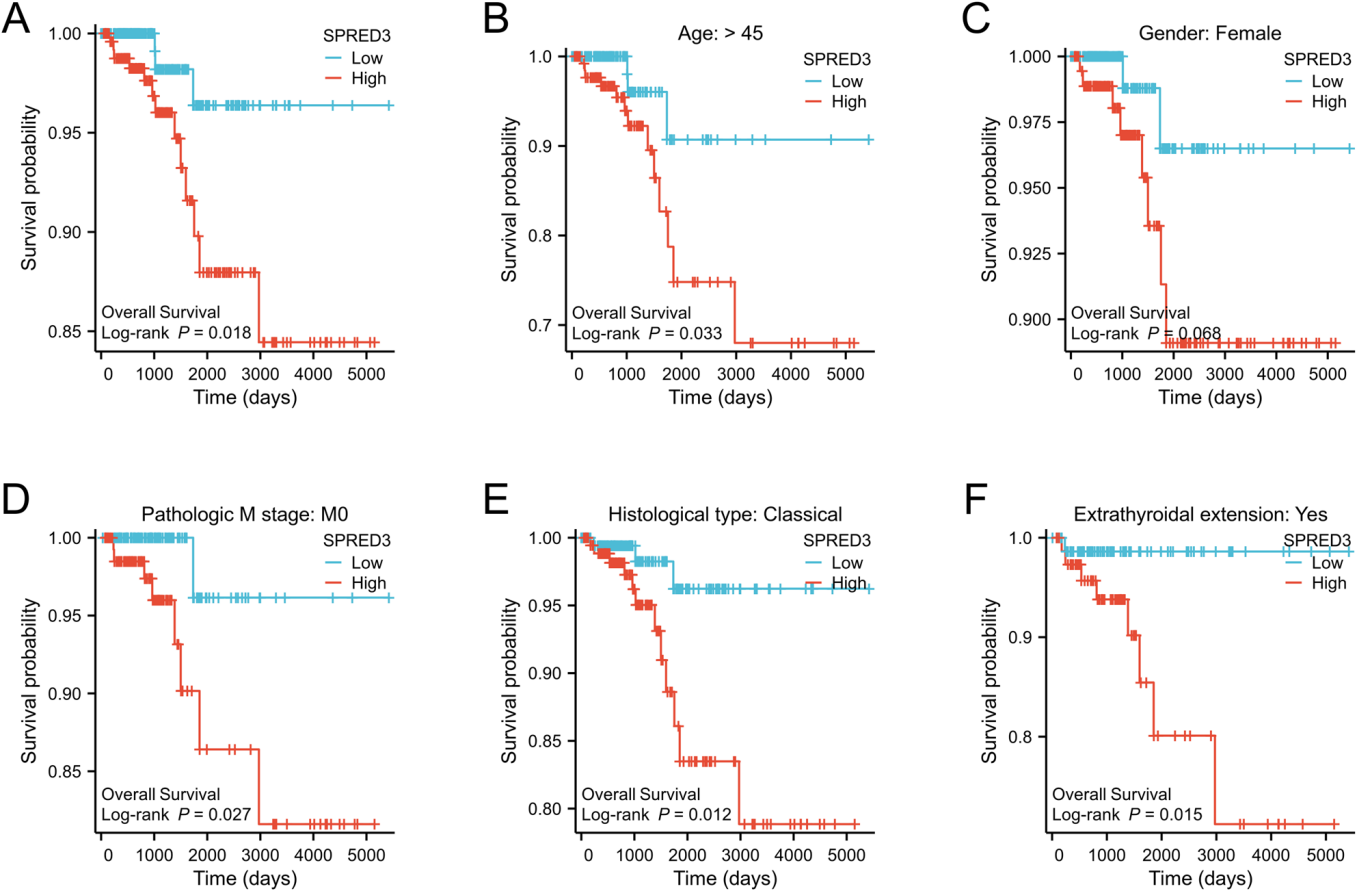
High SPRED3 expression predicts a significantly lower overall survival rate in THCA patients. (**A**) Kaplan–Meier curve illustrating the comparison of overall survival between subgroups of THCA patients with high/low SPRED3 mRNA expression; (**B**–**F**) comparison of overall survival among different subgroups of THCA patients based on age, gender, pathologic stages, histologic types, and extra thyroidal extension.

Subsequently, we evaluated the prognostic value of SPRED3 expression for THCA. Our analysis of the TCGA database revealed that patients with high SPRED3 expression exhibited a significantly lower overall survival rate than those with low SPRED3 expression, as evidenced by the Kaplan‒Meier curve results (Fig. 4A). These findings suggested that SPRED3 can serve as a valuable molecular marker for prognosis. Furthermore, we examined the correlation between SPRED3 expression and clinical prognosis in different subgroups of thyroid carcinoma patients. Notably, SPRED3 exhibited strong prognostic value in various subgroups, including patients older than 45 years, females, those with M0 stage disease, those with the classical subtype, and those with extrathyroidal extension (Fig. 4B–F). Next, we further investigated the correlation between SPRED3 expression and the clinical features of THCA patients documented in the TCGA database. The results showed that SPRED3 expression significantly differed according to clinical stage (T1/2 vs. T3/4), lymph node invasion (N0 vs. N1), primary tumor tissue type, external thyroid gland invasion, tumor residue, tumor location and previous thyroid history (Table 1). Furthermore, to assess the potential of SPRED3 as a prognostic marker for patients with thyroid carcinoma (THCA), RNAseq and clinical data from the TCGA-THCA datasets were obtained and organized using the STAR process. Univariate Cox regression analysis revealed that pathologic stage, distant metastasis, pathological stage, and SPRED3 expression (hazard ratio (HR) = 0.074; 95% CI 0.040–0.138; P < 0.001) were significant predictors of unfavorable outcomes in THCA patients. Multivariate Cox regression analysis demonstrated that even after adjusting for other factors, pathologic stage (HR; 95% CI; P < 0.001) and SPRED3 expression (HR; 95%; P < 0.001) were found to be independent predictors of poor prognosis in THCA patients (Table 2).

**Table 1:** Correlation analysis between SPRED3 and clinical indicators of thyroid carcinoma.

Characteristics	Low expression of SPRED3	High expression of SPRED3	*P* value
n	256	256	
Pathologic T stage, n (%)			
T1 and T2	166 (32.5%)	146 (28.6%)	0.069
T3 and T4	89 (17.5%)	109 (21.4%)	
Pathologic N stage, n (%)			
N0	131 (28.4%)	98 (21.2%)	< 0.001
N1	97 (21%)	136 (29.4%)	
Pathologic M stage, n (%)			
M0	137 (46.4%)	149 (50.5%)	1.000
M1	4 (1.4%)	5 (1.7%)	
Pathologic stage, n (%)			
Stage I and stage II	185 (36.3%)	155 (30.4%)	0.005
Stage III and stage IV	70 (13.7%)	100 (19.6%)	
Gender, n (%)			
Female	188 (36.7%)	185 (36.1%)	0.766
Male	68 (13.3%)	71 (13.9%)	
Age, n (%)			
≤ 45	122 (23.8%)	121 (23.6%)	0.929
> 45	134 (26.2%)	135 (26.4%)	
Histological type, n (%)			
Classical	157 (30.7%)	209 (40.8%)	< 0.001
Follicular and other and tall cell	99 (19.3%)	47 (9.2%)	
Residual tumor, n (%)			
R0	203 (45.1%)	189 (42%)	0.049
R1 and R2	22 (4.9%)	36 (8%)	
Extrathyroidal extension, n (%)			
Yes	64 (13%)	90 (18.2%)	0.026
No	178 (36%)	162 (32.8%)	
Primary neoplasm focus type, n (%)			
Multifocal	114 (22.7%)	119 (23.7%)	0.778
Unifocal	135 (26.9%)	134 (26.7%)	
Neoplasm location, n (%)			
Right lobe	124 (24.5%)	94 (18.6%)	0.011
Bilateral and isthmus and left lobe	131 (25.9%)	157 (31%)	
Thyroid gland disorder history, n (%)			
Normal	121 (26.7%)	165 (36.3%)	0.002
Lymphocytic thyroiditis and nodular hyperplasia and other, specify	96 (21.1%)	72 (15.9%)

**Table 2:** The prognostic value of SPRED3 in patients with thyroid carcinoma was determined through both univariate and multivariate Cox regression analyses.

Characteristics	Total (N)	Univariate analysis		Multivariate analysis	
		Hazard ratio (95% CI)	P value	Hazard ratio (95% CI)	P value
SPRED3	512		**0.013**		
Low	256	Reference		Reference	
High	256	4.079 (1.160–14.338)	**0.028**	3.948 (1.124–13.867)	**0.032**
Pathologic M stage	295		0.115		
M0	286	Reference			
M1	9	4.258 (0.909–19.952)	0.066		
Pathologic T stage	510		**0.033**		
T1 and T2	312	Reference		Reference	
T3 and T4	198	3.002 (1.041–8.656)	**0.042**	2.907 (1.009–8.375)	**0.048**
Pathologic N stage	462		0.547		
N0	229	Reference			
N1	233	1.405 (0.458–4.308)	0.552		

Significant values are given in bold.

### The expression level of SPRED3 in THCA tissues has exceptional prognostic value for patients with THCA

**Figure 5:**
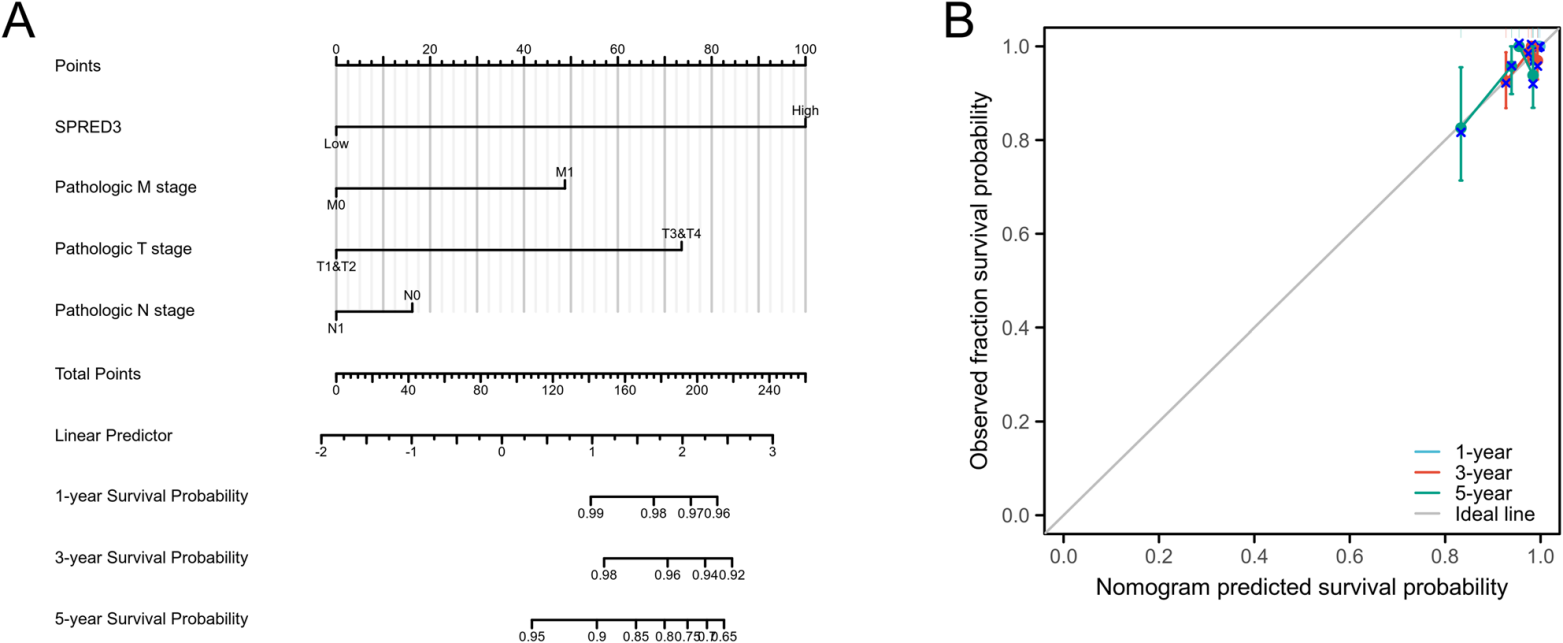
Nomogram curve was constructed to predict the probability of overall survival (OS) in patients at 1-, 3-, and 5-year intervals. The predictive factors included clinical stage, pathologic stage, and SPRED3 expression (**A**). Additionally, the nomogram was used to predict the 1-year OS in THCA population, as well as the 3-year and 5-year OS (**B**).

Additionally, a nomogram incorporating the pathologic stage and SPRED3 expression was constructed to predict the survival rates at 1 year, 3 years, and 5 years in THCA patients (Fig. 5A). These findings strongly suggest that SPRED3 can serve as an autonomous predictor of adverse outcomes in individuals with THCA. The combination of pathologic stage and SPRED3 expression was used to predict overall survival at 1-year, 3-year, and 5-year intervals in patients with THCA. A bias-corrected line was constructed to approximate the ideal curve, demonstrating complete concordance between the predicted outcomes and observed results at both the 3-year and 5-year time points (Fig. 5B). These findings underscore the robust clinical significance of SPRED3 as a prognostic marker for THCA patients.

### The role of SPRED3 in THCA was investigated through immune cell infiltration and gene enrichment analyses

**Figure 6:**
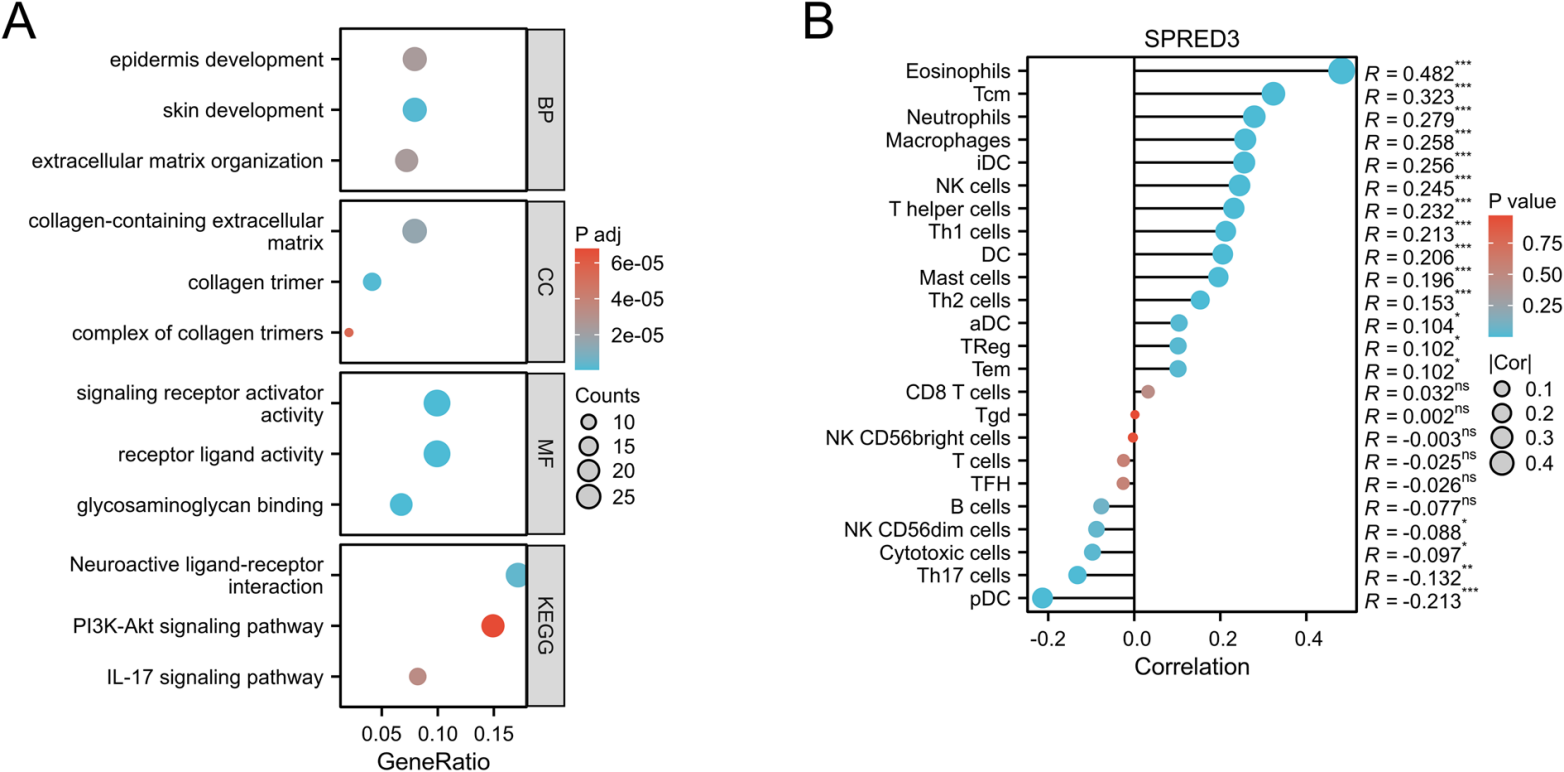
The differentially expressed genes were ranked based on the correlation factor, and subsequently, the gene list was subjected to GO/KEGG analysis for clustering purposes (**A**), and the correlation between infiltrated immune cells and SPRED3 expression levels in clinical samples of THCA patients was investigated.

To delineate potential gene regulatory networks associated with SPRED3, RNA-seq data corresponding to the genes were extracted from the TCGA public database and divided into high- and low-expression groups based on SPRED3 expression. Differentially expressed genes were identified using the DESeq2 package. Subsequently, gene enrichment analysis was performed, and the ggplot2 package was utilized to visualize the results of this analysis. Finally, GO/KEGG analysis was employed to classify the genes in the list. The gene set enrichment analysis shown in Fig. 6A indicated significant enrichment of several gene functional clusters, including extracellular matrix organization, epidermis development, signaling receptor activator activity, skin development, receptor ligand activity, glycosaminoglycan binding, and the neuroactive ligand‒receptor interaction pathway, in patients with high SPRED3 expression in the THCA cohort. Moreover, the IL-17 signaling pathway and PI3K-Akt signaling pathway were revealed to be significantly enriched. To investigate the immune-invasive characteristics of THCA patients with high SPRED3 expression, a correlation analysis between SPRED3 expression and immune cell infiltration was performed. As depicted in Fig. 6B, the experimental findings demonstrated a positive correlation between the expression level of SPRED3 and the relative abundance of 24 immune cell types within the tumor microenvironment. Notably, high SPRED3 expression was significantly associated with increased infiltration of eosinophils, central memory T cells (Tcm), neutrophils, macrophages, immature dendritic cells (iDCs), dendritic cells (DCs), T helper cells, Type 1 T helper cells (Th1), Type 2 T helper cells (Th2) cells, mast cells, regulatory T cells (Treg), effective memory T cells (Tem) and natural killer cells (NK). Furthermore, elevated SPRED3 expression was significantly negatively correlated with the infiltration of plasmacytoid dendritic cells (pDCs), Th7 cells, cytotoxic cells, NK CD56 dimeric cells and B cells.

### Immunohistochemical analysis revealed that SPRED3 expression was upregulated in thyroid carcinoma

**Figure 7:**
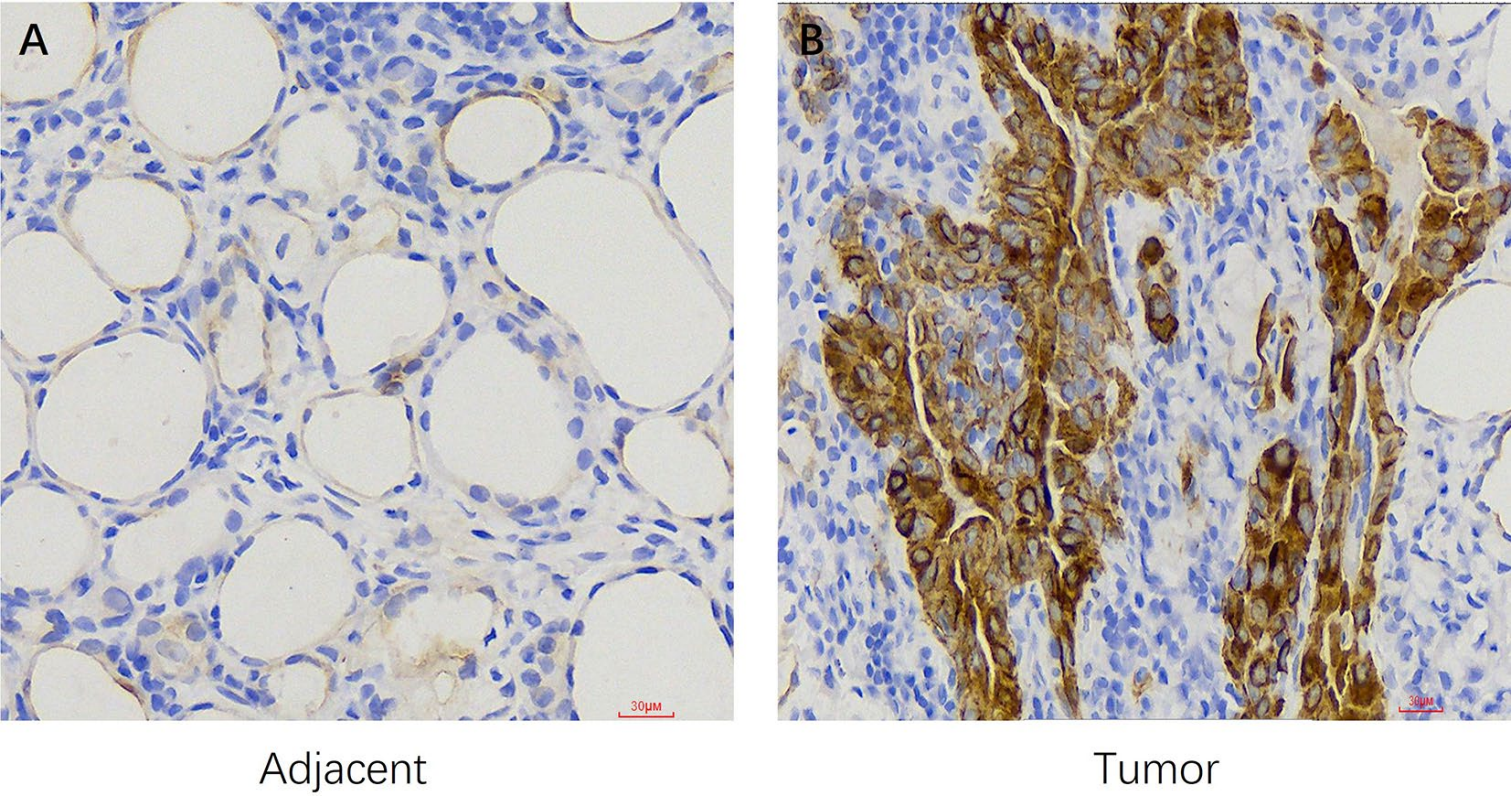
The protein expression level of SPRED3 was assessed in both the adjacent tissues (**A**) and the THCA tumor sample (**B**).

The bioinformatics data were subsequently validated through immunohistochemistry to confirm the expression of SPRED3 in thyroid carcinoma and corresponding paracancerous tissues. Paraffin-embedded tumor tissues were collected for the detection of SPRED3 expression. The results revealed high expression of SPRED3 in 34 out of 67 patients with thyroid carcinoma and in 13 of 67 paired para-cancerous normal tissues (P < 0.001; Fig. 7). Moreover, our data revealed a significant association between SPRED3 expression and clinical indicators, including pathologic T stage (P = 0.011), N stage (P < 0.001), and M stage (P = 0.002), and histological type (P < 0.001), residual tumor type (P = 0.021) and extrathyroidal extension (P = 0.019), in thyroid carcinoma patients (Table 3).

**Table 3:** Correlation between SPRED3 and clinical indicators in thyroid carcinoma patients.

Characteristics	Low expression of SPRED3	High expression of SPRED3	*P* value
n	33	34	
Pathologic T stage, n (%)			
T1 and T2	21	19	0.011
T3 and T4	12	15	
Pathologic N stage, n (%)			
N0	19	15	< 0.001
N1	14	19	
Pathologic M stage, n (%)			
M0	32	33	1.000
M1	1	1	
Pathologic stage, n (%)			
Stage I and stage II	24	21	0.002
Stage III and stage IV	9	13	
Histological type, n (%)			
Classical	20	28	< 0.001
Follicular and other and tall cell	13	6	
Residual tumor, n (%)			
R0	31	28	0.021
R1 and R2	2	6	
Extrathyroidal extension, n (%)			
Yes	7	12	0.019
No	26	22	

### Flowchart depicting the assessment of SPRED3 expression and its clinical significance in thyroid carcinoma

**Figure 8:**
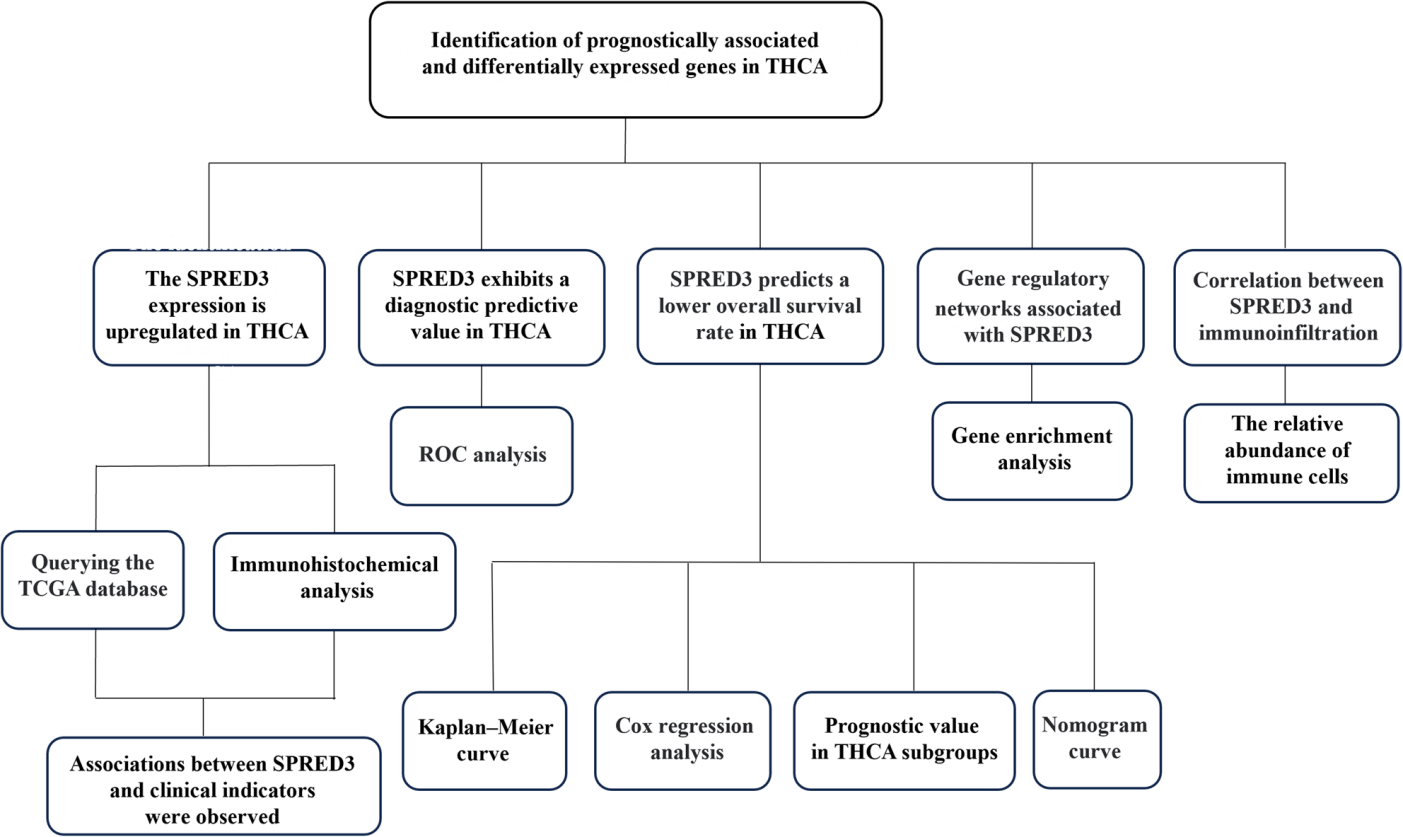
The flowchart depicting the assessment of SPRED3 expression and its clinical significance in thyroid carcinoma.

To enhance the clarity of our research concepts and findings, we constructed a flow chart to visually present the ideas and results outlined in this article (Fig. 8).

## Discussion

The present study employed bioinformatics analysis of RNA-seq data from TCGA clinical samples to comprehensively and meticulously assess SPRED3 expression, aiming to investigate its correlation with clinicopathological features and survival outcomes and its functional role in THCA development. These findings indicate that SPRED3 is significantly upregulated in tumor samples and may play a regulatory role in THCA progression. These results suggest the potential utility of SPRED3 as a biomarker for early diagnosis and prognosis prediction in THCA patients, particularly within specific disease stages, pathological classifications, and metastatic subgroups. Furthermore, immunohistochemical analysis of tumor tissues confirmed the bioinformatic analysis findings. The current findings suggest that the detection of SPRED3 expression could be employed as a practical approach to differentiate individuals at high risk of THCA progression, distal organ metastasis, and poor survival outcomes, thereby holding immense potential for clinical application.

Elucidation of the mechanism underlying distal metastasis in malignant tumors and identification of potential targets for tumor therapy are crucial and fundamental aspects of gene therapy. Sprouty-related EVH1 domain-containing proteins (SPREDs) are a class of membrane proteins^21^. Studies have demonstrated that SPREDs can negatively regulate the Ras-Raf-ERK signaling pathway, thereby inhibiting tumor development^22^. While all the SPRED variants can negatively regulate the RAS/MAPK pathway, it is worth noting that unlike SPRED1 and SPRED2, SPRED3 exhibited significantly weaker ERK-inhibitory activity due to its lack of a functional KBD^23^. Currently, most studies on SPREDs in cancer have focused on SPRED1 and SPRED2, and several studies have reported that SPRED1 deficiency is an important factor in the development of myeloid leukemia^24^. SPRED2 can regulate the proliferation and apoptosis of chronic myelogenous leukemia and hepatoma cells and inhibit the growth of tumors by inhibiting the RAS/MAPK pathway^25^. In addition, in contrast to the decreased expression of SPRED1 and SPRED2 in tumors^15^, this study revealed that SPRED3 expression was significantly upregulated in THCA and was associated with poor patient prognosis. However, the precise role of SPRED3 in tumorigenesis, development, and metastasis and the related mechanisms remain elusive. In this study, we validated the diagnostic value of SPRED3 in a specific subgroup of patients with thyroid carcinoma (THCA) through Kaplan‒Meier curve analysis. Notably, SPRED3 has emerged as a promising molecular marker for prognostic evaluation. Furthermore, our findings demonstrated the significant prognostic value of SPRED3 across various subgroups within the THCA patient population, including those aged > 45 years, females, M0 stage patients, those with classical grade tumors, and those with extrathyroidal invasion. Importantly, both univariate and multivariate Cox regression analyses consistently revealed that SPRED3 expression was an independent predictor of unfavorable prognosis in THCA patients even after adjusting for other confounding factors. Further analysis of RNA-seq data between high- and low-SPRED3 THCA patients was also conducted to screen for significantly differentially expressed genes and functional gene clusters associated with high SPRED3 expression. We hypothesized that SPRED3 plays a tumorigenic role in THCA partly through the activation of the PI3K/Akt and IL17 signaling pathways. In contrast to the current findings, no association between SPRED3 and the Ras-Raf-ERK signaling pathway was observed in THCA cells. To our knowledge, this study is the first to propose a correlation between SPRED3 and the PI3K/Akt and IL17 signaling pathways. The PI3K/Akt signaling pathway facilitates cellular metabolism and proliferation, contributing to cancer development, chemotherapy resistance, and angiogenesis regulation^26^. The cytokine IL-17 activates the NOTCH signaling pathway^27^, thereby participating in cellular proliferation and regulating the expression of inflammatory cytokines^28^. These signaling pathways play a crucial role in the progression of cancer, including THCA^29^. Furthermore, IL-17 has been demonstrated to interact with the PI3K/AKT signaling pathway^30^. Research has indicated that IL-17 inhibits Hep-2 cell apoptosis by suppressing the Fas/Fas L signaling pathway through modulation of the PI3K/AKT cascade^31^. Additionally, IL-17 upregulates cyclin D2 to activate the PI3K/Akt signaling pathway and promote DLBCL cell growth^32^. Based on the aforementioned findings, further investigations should be conducted in the future to explore the interplay between SPRED3, the PI3K/Akt pathway, and the IL17 signaling pathway. This research may provide novel insights for therapeutic development targeting high-risk THCA patients.

Additionally, RNA-seq analysis was employed in this study to determine the correlated between SPRED3 expression and the infiltration of different immune cell types. The present research revealed significant positive correlations between eosinophil, central memory T cell, neutrophil, macrophage, immature dendritic cell, and NK cell infiltration and SPRED3 expression in THCA patients. Moreover, increased SPRED3 expression is negatively associated with the infiltration of plasmacytoid dendritic cells and Th7 cells as well as cytotoxic cells and B cells. Immunotherapy has demonstrated promising outcomes in various malignancies, including THCA^33^. Therefore, further investigations are warranted to explore the impact of SPRED3 on immune cell infiltration in patients with THCA. It is worth exploring whether THCA patients with high SPRED3 expression can benefit from immunotherapy. Additionally, we conducted an immunohistochemical analysis to validate the findings observed through gene sequencing and discovered that, compared to that in paracancerous tissues, the expression of SPRED3 was upregulated in THCA tissues.

In our study, we were able to establish a regulatory network related to SPRED3. Therefore, SPRED3 may have negative implications for the prognosis of THCA patients and could be linked to immune cell infiltration in tumors. Furthermore, in addition to the various commonly used bioinformatics analysis methods and online tools, the association between SPRED3 and THCA was validated through immunohistochemical analysis. Moreover, the correlations between SPRED3 and clinical indicators in patients with thyroid carcinoma were further substantiated through the collection of clinicopathological data. Finally, unlike the findings of previous reports linking SPRED3 with ERK signaling pathways^34^, our study suggested that SPRED3 may exert its effects through the PI3K/Akt and IL17 signaling pathways. However, the recognition of the inherent limitations in this study is of utmost importance. To validate the prognostic value of SPRED3, a larger sample of patients with thyroid cancer (THCA) is needed. Furthermore, additional research through molecular biology experiments is necessary to substantiate any potential correlation between SPRED3 and the PI3K/Akt and IL17 signaling pathways. Moreover, advancement of interaction prediction research in various fields of computational biology could lead to valuable insights into genetic markers and ncRNAs associated with thyroid carcinoma, such as the prediction of miRNA–lncRNA interactions^35–41^. The investigation of ncRNAs implicated in the functionality of SPRED3 could also be pursued as a prospective avenue in our forthcoming endeavors. Furthermore, advances in interaction prediction research across various domains in computational biology could provide invaluable insights into genetic markers and their associated diseases^42–44^. This method of predicting interactions could also be applied to the SPRED3 protein for interaction prediction.

In conclusion, this study demonstrated that SPRED3 is highly expressed in patients with THCA through high-throughput bioinformatics analysis. Subsequent analyses revealed a correlation between high SPRED3 expression and pathological and clinical features in THCA patients. Further investigations demonstrated significant upregulation of SPRED3 in various functional signaling pathways, including the PI3K/Akt and IL17 signaling pathways, as well as correlations with immune cell infiltration. These findings suggest that SPRED3 is a potential diagnostic and prognostic marker for managing THCA patients.

## Methods

### Data collection

The gene expression data and clinical information utilized in this study were obtained from The Cancer Genome Atlas (TCGA) and included gene expression profiles and associated clinical data from 571 patients with thyroid carcinoma and 59 healthy individuals. The RNAseq data in TPM format were obtained from The Cancer Genome Atlas (TCGA) database (https://portal.gdc.cancer.gov). Differentially expressed mRNAs were identified based on an absolute log2-fold change (|logFC|) > 1.5 and an adjusted p value (*P*.adj) < 0.05, and we used R language to identify mRNAs coexpressed with the target gene. All procedures for data processing adhered to the principles outlined in the Declaration of Helsinki.

### Univariate and multivariate Cox regression analyses

The associations between SPRED3 expression and tumor grade, pathological stage, TNM stage, and prognosis were investigated using univariate and multivariate Cox regression analyses. Factors unrelated to prognosis were excluded from the analysis. Additionally, we used the rms package and survival receiver operating characteristic (ROC) package to develop a nomogram that accurately reflects the 1-, 3-, and 5-year survival rates of patients with THCA. Based on the expression level of the SPRED3 gene, THCA patients were stratified into high- and low-expression groups. The differences in overall survival (OS) between these groups were explored using the Kaplan‒Meier method and a two-sided log-rank test. To evaluate the performance of our constructed nomogram, calibration curve analysis and calculation of the consistency index (C-index) were conducted.

### Functional enrichment analysis

The THCA patients were stratified into high- and low-expression groups based on SPRED3 gene expression levels using Gene Ontology (GO) and Kyoto Encyclopedia of Genes and Genomes (KEGG) enrichment analyses. RNA-seq data obtained from The Cancer Genome Atlas (TCGA) database were subjected to R analysis to investigate the associations between SPRED3 expression and pathological and clinical parameters in THCA patients. Signaling pathways with an NES > 1.5 and P < 0.05 were considered significantly enriched. The results of the enrichment analysis were visualized using the ggplot2 package.

### Immune cell infiltration assessment using ssGSEA

The infiltration level of each specific immune cell type in clinical THCA samples was determined using the corresponding gene expression profiles, and immune infiltration analysis was performed via single-sample gene set enrichment analysis (ssGSEA) via the "GSVA" R package. Furthermore, Spearman correlation analysis was used to investigate the association between immune cell infiltration and SPRED3 expression, while the Wilcoxon rank sum test was used to explore the correlation between the infiltration levels of different immune cell types and SPRED3 expression.

### Immunohistochemistry

Immunohistochemical analysis was also conducted to assess the expression levels of SPRED3 in paraffin-embedded biopsy samples collected from treatment-naive patients diagnosed with primary THCA between January 2019 and December 2022. The experimental protocol was established using rabbit polyclonal antibodies specific for human SPRED3 (GTX85375, GeneTex, China). Negative control sections were treated with isotype-matched antibodies at an equivalent dilution as that used for the primary anti-human SPRED3 antibody. The positive localization of SPRED3 at both the cellular membrane and the cytoplasmic region served as a criterion for determining its presence. A semiquantitative approach involving a combination of the percentage of positively stained tumor cells and staining intensity, as previously described^45^, was utilized for classifying and scoring protein expression levels.

### Statistical analysis

Statistical processing was performed using R version 3.5.1. Spearman expression differences between THCA tissue and normal thyroid tissue were tested by the Wilcoxon rank sum test, and differences in expression between paired samples were tested by paired t tests. The diagnostic value of SPRED3 expression was evaluated by receiver operating characteristic (ROC) curve analysis. The value of SPRED3 as a prognostic factor in patients with THCA was detected by univariate and multivariate Cox regression analyses. Overall survival (OS) in the SPRED3 high-expression group and low-expression group was evaluated by the Kaplan‒Meier method and log-rank test, and a Cox proportional hazard regression model was used for multivariate analysis. The association between SPRED3 expression and clinical indicators in thyroid carcinoma patients was investigated using a chi-square test, with P < 0.05 indicating statistical significance.

### Ethics approval and informed consent

The Ethics Committee of Zibo Central Hospital has granted approval for this study. The research program adheres to the scientific and ethical principles outlined in the Declaration of Helsinki, and written informed consent has been obtained from all patients.

## Data Availability

The datasets used and/or analyzed during the present study are available from the corresponding author on reasonable request.
